# Exploring the *Staphylococcus aureus* Gyrase Complex and Human Topoisomerase: Potential Target for Molecular Docking and Biological Studies with Substituted Polychloroaniline

**DOI:** 10.1021/acsomega.3c05970

**Published:** 2023-11-16

**Authors:** Richa Tomar, Paratpar Sarkar, Vivek Srivastava, Pankaj Gupta, Sumira Malik, Azmat Ali Khan, Murtaza Tambuwala

**Affiliations:** † Department of Chemistry & Biochemistry, Sharda School of Basic Sciences & Research, Sharda University, Greater Noida 201310, India; ‡ Department of Chemistry and Biochemistry, School of Basis Sciences and Research, Sharda University, Greater Noida 201310, U.P., India; § Amity Institute of Biotechnology, Amity University, Jharkhand, Ranchi 834001, India; ∥ Pharmaceutical Biotechnology Laboratory, Department of Pharmaceutical Chemistry, College of Pharmacy, King Saud University, Riyadh 11451, Saudi Arabia; ⊥ Lincoln Medical School, University of Lincoln, Brayford Pool Campus, Lincoln LN6 7TS, U.K.; ▲ Department of Biotechnology, University Center for Research & Development (UCRD), Chandigarh University, NH-05 Chandigarh-Ludhiana Highway, Mohali, Punjab 140413, India

## Abstract

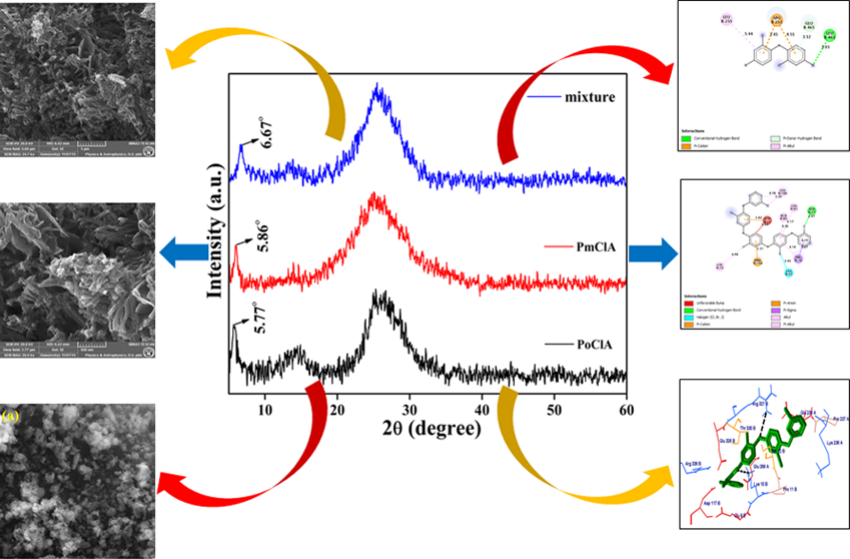

This paper targets the nuclease activity of polymeric chemical compounds toward bacterial genomic DNA and also elucidates their probable drug-like properties against the enzymes bacterial gyrase complex and human topoisomerase. Poly-*o*-chloroaniline, poly-*m*-chloroaniline, and poly-*o*,*m*-chloroaniline were synthesized by a chemical oxidation method. The structure of the polymers was characterized by the powder X-ray diffraction pattern, which suggested the ordered structure of the polymer, where the parallel and perpendicular periodicities of the polymeric chain were arranged systematically. The molecular transition of polymers was determined by a UV–visible spectrum study. A polymeric arrangement of the molecule can be seen in scanning electron microscopy (SEM) images. Among the three polymers chosen for the biological study and molecular docking studies, poly-*m*-chloroaniline showed more affinity to bind against both the selected targets (HT IIIb TB and SAGS) in comparison to the ortho- and ortho-meta substituents of polyaniline. The biophysical interaction analysis is in line with molecular docking, which shows that poly-*m*-chloroaniline forms many different categories of interactions and binds very strongly with the selected targets. The synthesized and tested molecules have potential nuclease activity, which is well aligned with molecular docking studies against the bacterial gyrase complex and human topoisomerase.

## 1 Introduction

DNA is a naturally occurring steady polymer with a predicted half-life of ∼130,000 years under physiological conditions under spontaneous hydrolysis.^1^ DNA cleaving agents are charged with the recognition of their potential applications in the area of biological sciences and as therapeutic agents. For the efficient cleavage of DNA by either hydrolytic or oxidative pathways,^2^ many metal complexes have been explored. Due to their broad spectrum of biological activities, heterocyclic compounds are dominant in the area of medicinal chemistry.^3^ The most feasible main cellular target for platinum drugs is genomic DNA. It has been identified especially for cisplatin that significant antitumor activity comes out from the cross-linking of intrastrain and bending of DNA.^4^ Therefore, targeting DNA through drugs remains in the spotlight, and the action of compounds regarding cancer cells selectively over healthy cells is attracting more attention in research.^5,6^ Conducting polymers have attracted immense interest due to their various physical and chemical properties and their numerous applications.^7−9^ Among various conducting polymers, aryl amine polymer (PANI) is a semiflexible conducting polymer of the organic semiconductor family, which has attractive intensive interest due to its remarkable potential applications such as biomedical, photovoltaic cells, supercapacitors, fuel cells, biosensors, electrocatalysis, photocatalysis, and adsorption of wastewater.^10−12^ The major advantages of this polymer are its high electronic conductivity, high environmental stability, and easy synthesis. The major disadvantage of the PANI is its solubility, which is improved by different substitutions in the benzene ring of aniline.^13^ PANI and the substituted polyaniline with an electron-donating group (poly-m-aminophenol) show a high degree of crystallinity and a more ordered structure. However, if attached with the I^–^ group, the polymer shows slower polymerization, but, exceptionally, F^–^ shows high crystallinity because of its small size and gives the opportunity for molecular interaction of the polymer segment.^14^ It was found that the substituent group of aniline affects not only the polymerization reaction but also the properties of the polymers obtained. DNA templates fabricated with polyaniline nanowires on Si surfaces prevent the accumulation of DNA produced by shielding of charges on DNA when polyaniline-DNA complexes are formed in solution.^15^ Polyanalines responsible for the antibacterial behavior of different microorganisms, namely, Gram-positive (like *S. aureus*, *Bacillus subtilis*, *Streptococcus pyogenes*, and *Streptococcus mutans*) and Gram-negative (*Salmonella Typhi*, *Klebsiella pneumoniae*, *Escherichia coli*, and *Pseudomonas aeruginosa*) bacteria, were chosen because of their pharmacological significance.^16^

Molecular docking is the process of allowing different synthesized compounds to bind to different amino acid residues of different protein and enzyme targets using an *in silico* approach.^17^ Molecular docking simulation is a very popular and well-established computational approach and has been extensively used to understand the molecular interactions between a natural organic molecule (ideally taken as a receptor), such as an enzyme, protein, DNA, or RNA, and a natural or synthetic organic/inorganic molecule (considered as a ligand). But the implementation of docking ideas to synthetic organic, inorganic, or hybrid systems is very limited with respect to their use as a receptor despite their huge popularity in different experimental systems. In this context, molecular docking can be an efficient computational tool for understanding the role of intermolecular interactions in hybrid systems, which can help in designing materials on mesoscale for different applications.^18^

In this study, we precisely examine the nuclease activity of the chemical compound poly-*o*-chloroaniline and poly-*o*,*m*-chloroaniline complexes toward bacterial genomic DNA. Several previous reports have noted the possible role of DNA cleavage catalysis chemistry.

## 2 Experimental Section

### 2.1 Synthesis of Poly-*o*-chloroaniline (PoClA)

Five mL of *o*-chloroaniline (99.0%, Thomas Baker) was mixed with a solution containing 35 mL of 2 M HCl (Merck, 99.9%) and 165 mL of water. To this, 0.275 g of FeCl_2_·2H_2_O (CDH, 99.0%) was added (keeping the molar ratio of metal salt:*o*-chloroaniline as 1:25) together with 6 v/v % of 10 mL H_2_O_2_ (Merck, 30 v/v %). The mixture was stirred at room temperature for 24 h. A dark greenish black-colored solid was formed after filtering, and the reaction mixture was mixed with 1:1 HCl:H_2_O and methanol.

### 2.2 Synthesis of Poly-*m*-chloroaniline (PmClA)

Five mL of *m*-chloroaniline (99.0%, Thomas Baker) was mixed with a solution containing 35 mL of 2 M HCl (Merck, 99.9%) and 165 mL of water. The same procedure as for poly-*o*-chloroaniline was followed.

### 2.3 Synthesis of Poly-*o*,*m*-chloroaniline (PomClA)

2.5 mL of *o*-chloroaniline (99.0%, Thomas Baker) and 2.5 mL of *m*-chloroaniline (99.0%, Thomas Baker) were mixed with a solution containing 35 mL of 2 M HCl (Merck, 99.9%) and 165 mL of water. The same procedure was followed as in the case of poly-*o*-chloroaniline.

### 2.4 Characterization Methods

Powder X-ray diffraction (PXRD) patterns were recorded using a high-resolution X ‘Pert PRO with Cu Kα radiation (45 kV and 40 mA) at a speed of 2°/min over the range 10–50°. FTIR spectra were recorded using a PerkinElmer 2000 Fourier-transform infrared spectrometer with KBr disks. The surface morphology of the obtained powder was investigated by scanning electron microscopy (SEM) and energy-dispersive X-ray spectroscopy using a JEOLT330 microscope. The UV–visible absorbance data of samples in solution form (in dimethylformamide, DMF) were collected over the spectral range 200–800 nm using a UV–visible spectrophotometer (PerkinElmer Lambda 35).

### 2.5 Molecular Docking Study and Biophysical Interaction Study

Patchdock server is a molecular docking software used for molecular docking experiments.^19^ The human cancer target protein chosen was Human Topoisomerase III b topo domain (HT IIIb TB) with PBD Id: 5GVC, which is a DNA/RNA topoisomerase that has a well-defined role in epigenetic and translational control of gene expression, and the bacterial target protein *Staphylococcus aureus* gyrase complex (SAGS) with PBD Id: 6FM4 was selected for the molecular docking study. DNA gyrase is an essential topoisomerase that supercoils DNA through a process of strand breakage/resealing and DNA wrapping. Both these proteins were downloaded from the RSCB Protein Data Bank in PDB format. All of the synthesized ligands and the complexes formed from these ligands were drawn using Chem Sketch and the mol files generated were then converted to PDB format for molecular docking against the two target proteins selected against the selected proteins. Docking files were then visualized, and different interactions between the ligands and targets were identified and analyzed using a visualizing software known as Discovery Studio. The results of molecular docking experiments were depicted in the form of different biophysical interactions, viz, attractive van der Waals energy (AvdW), repulsive van der Waals energy (RvdW), atomic contact energy (ACE), and solvation energy. The total energy of the interactions also has been depicted in the form of total binding energy (TBE).

### 2.6 DNA Cleavage Study

DNA cleavage experiments of the prepared compounds were performed against genomic DNA in a reaction buffer containing 100 mM NaCl, 50 mM Tris-HCl, and 10 mM MgCl_2_. The reactions were incubated at 37 C for 1 h and stopped by adding a DNA loading dye [0.25% bromophenol blue (w/v), 0.25% xylene cyanol (w/v), and 30% glycerol (v/v)]. Samples were resolved on 1% agarose gel in 1X-TAE (40 mM Tris-20 mM Acetate-1 mM EDTA) running buffer at 90 V for 1 h. The nuclease efficiency was measured by matching the band patterns obtained from untreated and treated genomic DNA in UV light using ImageJ software, and a graph was plotted in Excel.

## 3 Results and Discussion

### 3.1 Characterization Study

Polymer is often amorphous in nature. However, the salt form of polymer could exist in a semicrystalline state. For PANI, powder X-ray data in the 2θ range of 20° and above are usually reported. Only a few studies have reported 2θ values below 20° for PXRD and an elaborated crystallographic orientation of PANI.

**Figure 1:**
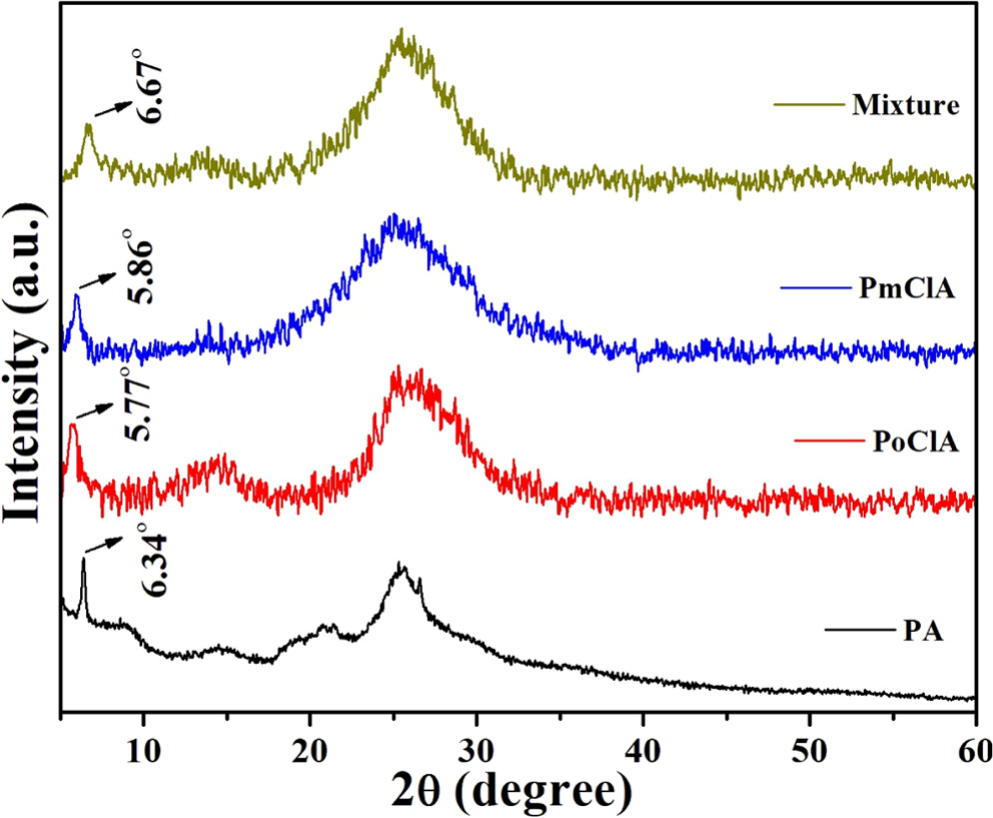
Powder X-ray diffraction patterns of polyaniline, poly-*o*-chloroaniline, poly-*m*-chloroaniline, and copolymerization of *o*,*m*-chloroaniline.

In the recent report, reflection around the 2θ range of 5.7–6.7° has been observed. Reflection values at 2θ of approximately around 6° and between 20 and 30° were observed in the PXRD pattern as shown in Figure 1, suggesting the ordered structure of the polymer.^20^ The reflection in the 2θ range of 20–30° has been attributed to the parallel and perpendicular periodicity of the polymeric chain.^20−22^ The highly ordered structure of chlorine-substituted PANI in our sample can result in the formation of linear 1D emeraldine salts arising from the doping of HCl in chlorine-substituted PANI.

**Figure 2:**
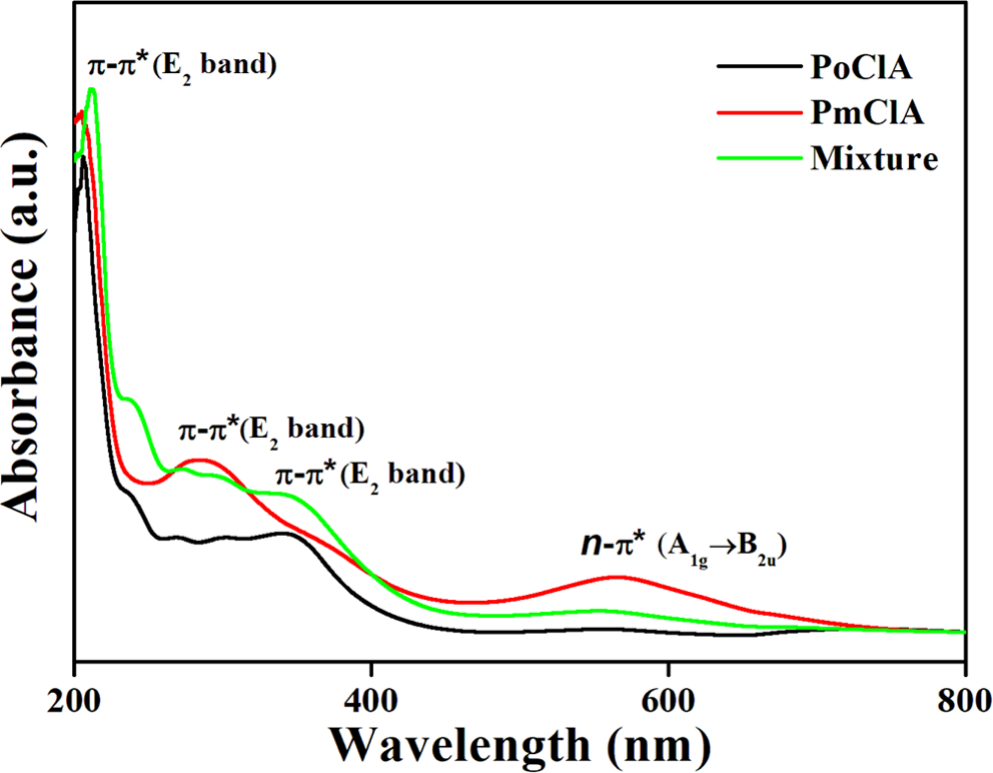
UV–visible spectra of poly-*o*-chloroaniline, poly-*m*-chloroaniline, and copolymerization of *o*,*m*-chloroaniline.

The UV–visible spectrum of the polymer is presented in Figure 2. The band at 244–380 nm corresponds to the π–π* electronic transition (E_2_-Band) of the phenyl ring in the polymer backbone and the band at around 500 nm corresponds to the B-band n-π* transition (A_1g_ → B_2u_) due to interband charge transfer associated with excitation of benzenoid to quinoid moieties.^23^

**Figure 3:**
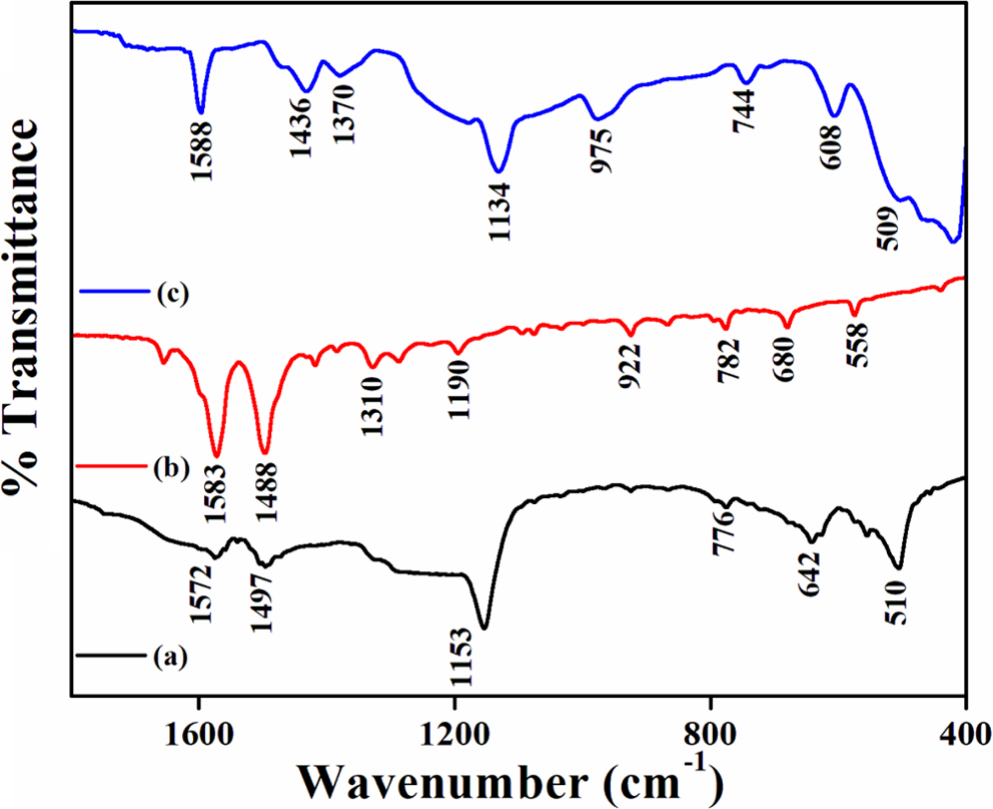
FTIR spectra of (a) poly-*o*-chloroaniline, (b) poly-*m*-chloroaniline, and (c) copolymerization of *o*,*m*-chloroaniline.

FTIR spectra of the polymers are given in Figure 3. The bands at 1588, 1488, 1310, 1190, 922, 782, 680, and 558 cm^–1^ correspond to –C=C– for a benzene ring (π–π interaction), Ar–N– (π–π interaction), skeletal vibration of C–N (aryl NH or aryl NH_2_), C–N stretching vibration or in-plane CH deformation in the aromatic ring, and out-of-the-plane CH deformation showing a 1,4-disubstituted benzene ring and=C–H and Ar–Cl interactions of the polymer.^1^

**Figure 4:**
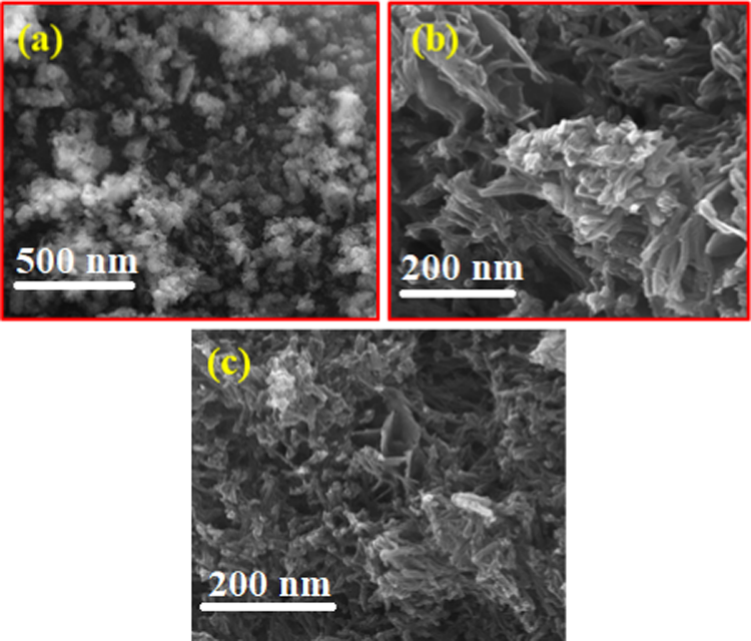
SEM images of (a) poly-*o*-chloroaniline, (b) poly-*m*-chloroaniline, and (c) copolymerization of *o*,*m*-chloroaniline.

Figure 4 shows the SEM images of the polymers. Some agglomerated chain-type morphology has been observed in poly-*o*-chloroaniline. A layered-type morphology has been shown in poly-*m*-chloroaniline, which has also been observed in the case of poly-*o*,*m*-chloroaniline.^1^

### 3.2 Molecular Docking and Biophysical Interaction Study

**Figure 5:**
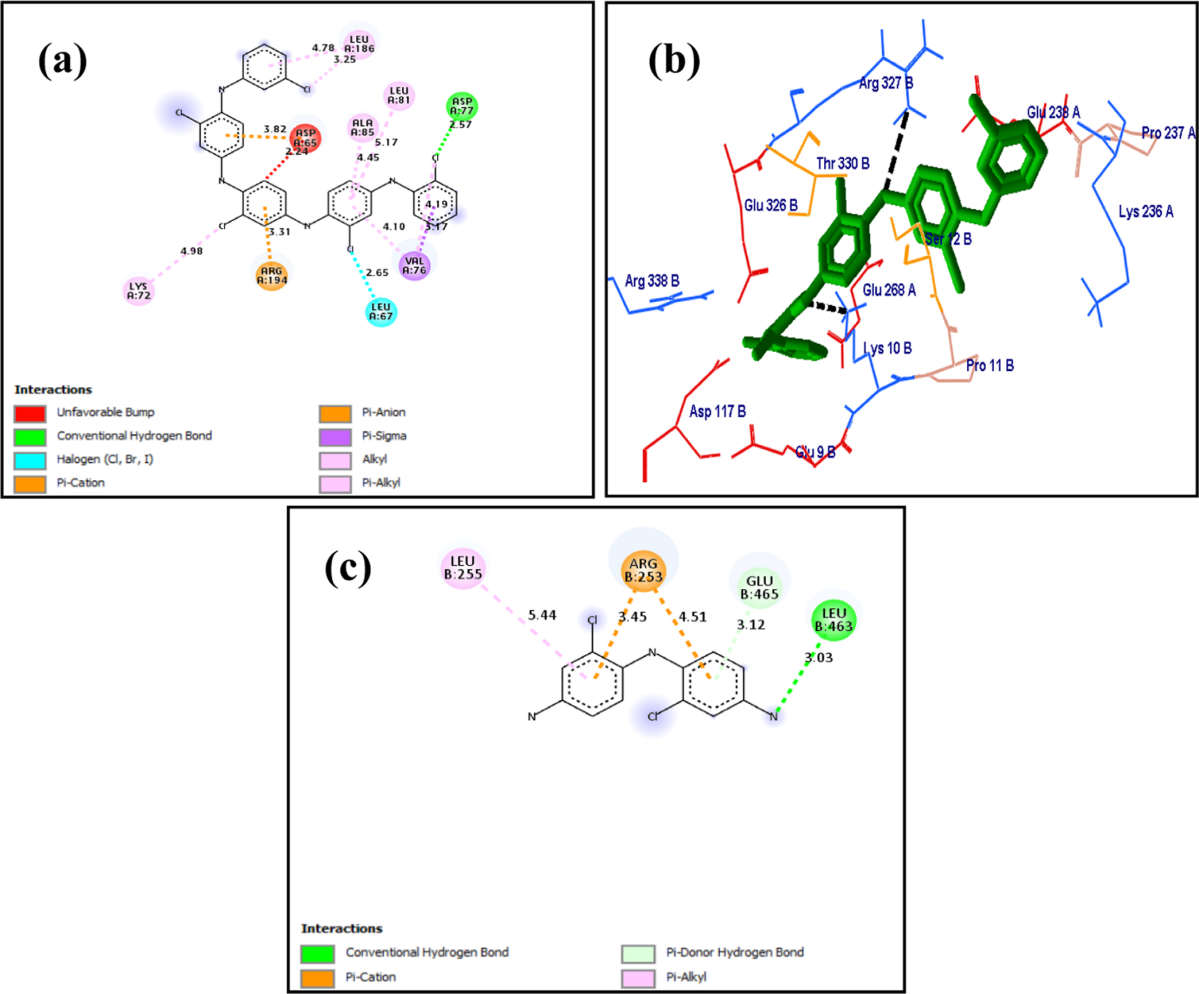
Molecular docking visualization of Human Topoisomerase III b topo domain (HTIIIbTB) with different substituted compounds of polyaniline: (a) poly-*m*-chloroaniline vs HTIIIbTB with a total binding energy of −48.93 kcal/mol, (b) poly-*o*-chloroaniline vs HTIIIbTB (−35.71 kcal/mol), and (c) poly-o-*m*-chloroaniline vs HTIIIbTB (−37.11 kcal/mol).

In the visualization study and molecular docking analysis (Figure 5(a)), the ligand (poly*-m*-chloroaniline) was found to make a Pi alkyl bond with Leu 168, Leu 81, and Val 76 of the target protein, HTIIIbTB. Similarly, the chlorine atom of the ligand makes an alkyl interaction with Leu 168. It is also seen that a conventional hydrogen bond is formed between the chlorine atom of the ligand and the hydrogen atom of Asp 77 of the target protein. The poly-*o*-chloroaniline in Figure 5(b) makes two conventional hydrogen bonds with Asp 327 and Glu 268 of the target protein, HTIIIbTB. Further, poly-*o*,*m*-chloroaniline (Figure 5(c)) makes two Pi cation interactions with Arg 253, Pi alkyl interaction with Leu 255, and a conventional hydrogen bonding with Leu 463. Ten best poses of molecular docking study of poly-*m*-chloroaniline against HTIIIbTB were chosen (Table 1). Each of the ten best poses gave one solution, and each solution had different biophysical interactions, viz., TBE, AvdW, RvdW, and ACE. In the same line of context, Tables 2 and 3 also depict different biophysical interactions of the other two synthesized ligands (poly-*o*-chloroaniline and poly-*o*,*m*-chloroaniline) against HTIIIbTB.

**Table 1:** Molecular Docking Analysis of the Top Ten Results: Biophysical Interaction of Poly-*m*-chloroaniline with Human Topoisomerase III B Domain^a^

name of solution	TBE (kcal/mol)	AvdW energy (kcal/mol)	RvdW energy (kcal/mol)	ACE (kcal/mol)
8	–48.93	–24.64	14.62	–15.55
10	–43.79	–18.49	4.68	–12.94
2	–42.81	–21.80	5.46	–10.32
6	–37.32	–19.93	3.58	–9.71
9	–34.21	- 16.36	8.59	–11.08
3	–33.34	–19.88	9.34	--8.00
5	–22.11	–17.94	8.61	–2.60
1	–21.03	–26.08	46.77	–14.17
4	64.43	–25.96	153.55	–11.92
7	550.70	–36.74	176.19	–10.59

a TBE is the total binding energy; AvdW is the attractive van der Waals force; RvdW is the repulsive van der Waals force; ACE is the atomic contact energy.

**Table 2:** Molecular Docking Analysis of Top Ten Results: Biophysical Interaction of Poly-*m*-chloroaniline with *S. aureus* Gyrase Complex

name of solution	TBE (kcal/mol)	AvdW energy (kcal/mol)	RvdW energy (kcal/mol)	ACE (kcal/mol)
5	--77.25	–30.80	10.64	–24.75
4	--65.20	--26.06	12.68	--23.02
6	--61.68	--28.63	21.13	--22.36
10	--58.65	--25.46	19.25	--22.55
3	--55.65	--21.95	5.28	--17.00
1	--53.15	--29.98	12.43	--11.64
2	--49.64	--26.21	9.69	--11.84
7	--27.52	--28.20	54.75	--18.55
8	--16.92	--12.36	5.37	--1.91
9	--39.87	--36.84	170.69	--26.34

**Table 3:** Molecular Docking Analysis of Top Ten Results: Biophysical Interaction of Poly-*o*-chloroaniline with 5GVC

name of solution	TBE (kcal/mol)	AvdW energy (kcal/mol)	RvdW energy (kcal/mol)	ACE (kcal/mol)
10	–35.71	–26.42	15.37	–15.42
5	–24.27	–16.26	15.84	–8.84
2	–17.24	–19.82	6.11	–3.43
7	–14.31	–12.58	7.19	–3.70
4	–9.60	–22.75	28.90	–6.47
3	–0.91	–6.73	3.07	–5.23
8	–0.12	–23.13	58.64	–9.68
6	5.18	–21.84	54.84	–6.02
9	95.49	–28.38	184.77	–7.89
1	347.99	–26.95	491.56	–3.30

**Figure 6:**
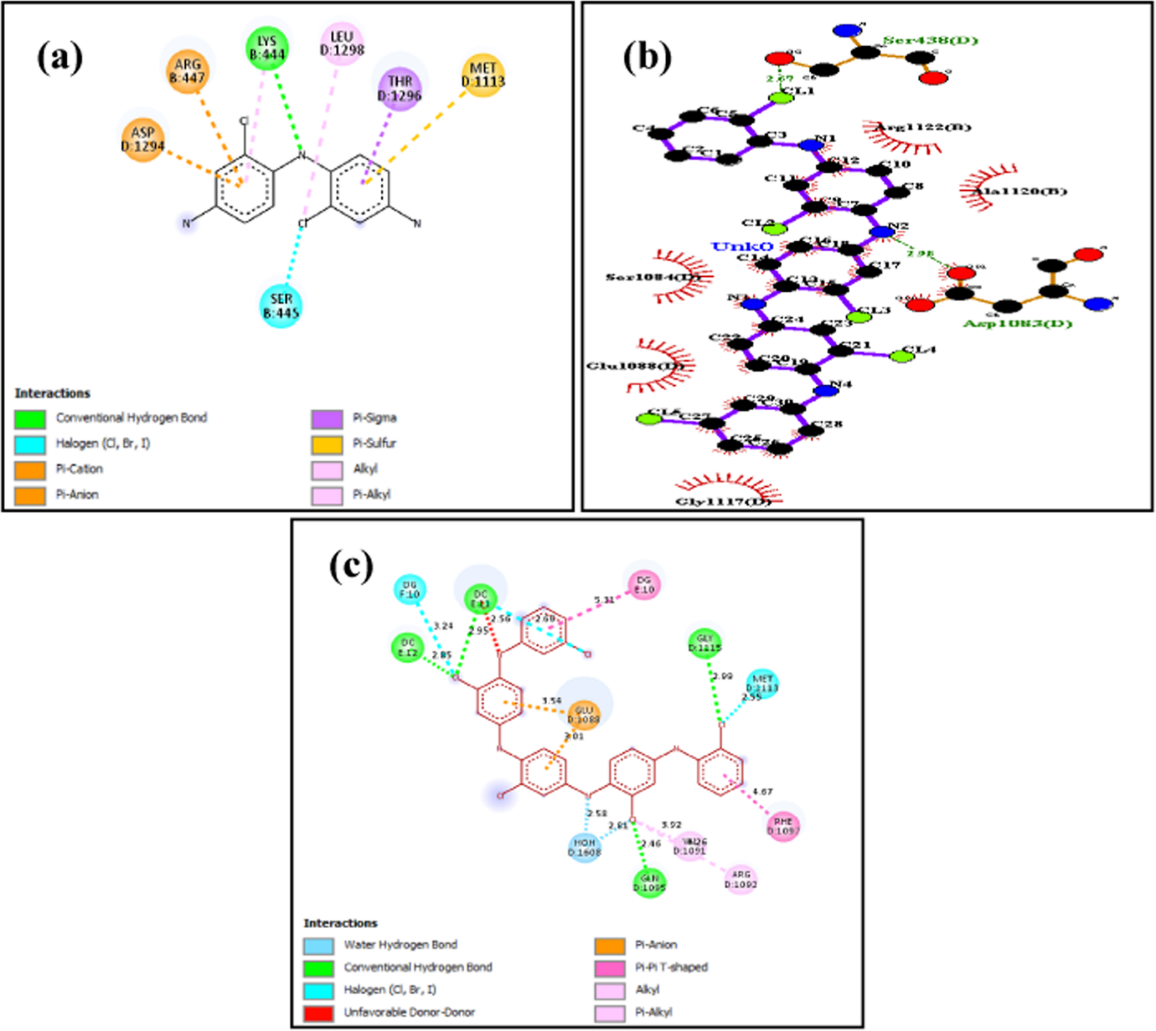
Molecular docking visualization of *S. aureus* gyrase complex (SAGS) with different substituted compounds of polyaniline: (a) poly-*o*,*m*-chloroaniline vs SAGS (−42.48 kcal/mol), (b) poly-*o*-chloroaniline vs SAGS (−72.17 kcal/mol), and (c) poly-*m*-chloroaniline vs SAGS (−77.25 kcal/mol).

The total binding energy released during the docking study shows that the compound poly-*m*-chloroaniline binds strongly with the target with a TBE of 48.93 kcal/mol followed by poly-*o*-chloroaniline (−35.71 kcal/mol) and poly-*o*,*m*-chloroaniline (37.11 kcal/mol). The results are depicted in Figure 5(a)–(c). Figure 6(c) depicts the molecular docking interaction of poly-metachloroaniline against SAGS. The compound makes a hydrogen bond with Gly 1115 and Gln 1095. The compound makes a Pi-Pi T-shaped interaction with PHE 1097 and two Pi-anion interaction with Gln 1088. Further results from Figure 6(a) depict the molecular docking results of poly-*o*,*m*-chloroaniline against SAGS.

The compound makes Pi cation interactions with Arg 447 and Asp 1294: Pi anion with Met 1113, Pi alkyl with Lys 444, alkyl with Lys 1298, and Pi sigma with Ala 1296. Conventional hydrogen bonding interaction occurs with Lys 444 and halogen interaction with Ser 445. The compound poly-*o*-chloroaniline against SAGS in Figure 6(b) shows a different result with only two conventional hydrogen bonding with Ser 438 and Asp 1083. Ten best poses of the molecular docking study of poly*-m*-chloroaniline against SAGS were taken for study (Table 4). Each of the ten best poses showed different biophysical interactions, viz., TBE, AvdW, RvdW, and ACE. In the same line of context, Tables 5 and 6 also depict different biophysical interactions of the other two synthesized ligands (poly-*o*-chloroaniline and poly-*o*,*m*-chloroaniline) against SAGS. The total binding energy released during the docking study shows that the compound poly-*m*-chloroaniline binds strongly with the target SAGS with a TBE of −77.25 kcal/mol, followed by poly-*o*-chloroaniline (−72.17 kcal/mol) and poly-*o*,*m*-chloroaniline (−42.48 kcal/mol). The molecular docking studies followed by biophysical interaction study confirm that poly*-m*-chloroaniline could bind more strongly with both the targets HTIIIbTB and SAGS out of the other two substituents of polyaniline. The compound can thus be taken further for various in vitro studies against different pathological conditions of both humans and bacteria.

**Table 4:** Molecular Docking Analysis of Top Ten Results: Biophysical Interaction of Poly-*o*-chloroaniline with 6FM4

name of solution	TBE (kcal/mol)	AvdW energy (kcal/mol)	RvdW energy (kcal/mol)	ACE (kcal/mol)
2	–72.17	–29.17	15.10	–25.22
3	–69.34	–31.04	11.95	–24.30
6	–65.12	–27.78	15.17	–23.36
4	–55.92	–26.84	12.07	–21.11
7	–52.91	–23.38	7.32	–15.2
10	–37.23	–21.00	18.97	–13.40
8	–36.72	–26.17	26.76	–18.23
9	–35.49	–22.94	23.90	–12.96
5	–32.79	–31.86	61.65	–28.24
1	5.49	–32.53	118.41	–30.40

**Table 5:** Molecular Docking Analysis of Top Ten Results: Biophysical Interaction of Poly-*o*,*m*-chloroaniline with Human Topoisomerase III b Domain

name of solution	TBE (kcal/mol)	AvdW energy (kcal/mol)	RvdW energy (kcal/mol)	ACE (kcal/mol)
8	–37.11	–15.82	3.67	–10.76
4	–31.52	–12.92	0.43	–8.62
5	–27.42	–10.60	4.23	–7.80
3	–24.91	–10.23	2.42	–7.97
10	–24.30	–13.73	0.27	–3.37
9	–19.55	–9.91	4.40	–6.11
7	–19.48	–12.17	4.55	--3.56
6	–15.52	–10.06	3.57	–9.00
2	–14.15	–13.39	10.40	–2.33
1	–11.21	–8.18	1.02	–0.65

**Table 6:** Molecular Docking Analysis of Top Ten Results: Biophysical Interaction of Poly-*o*,*m*-chloroaniline with *S. aureus* DNA Gyrase Complex

name of solution	TBE (kcal/mol)	AvdW energy (kcal/mol)	RvdW energy (kcal/mol)	ACE (kcal/mol)
9	–42.38	–16.15	0.00	–11.45
1	–41.54	–14.92	20.72	–13.60
6	–38.69	–14.42	1.25	–11.58
8	–37.76	–13.96	2.55	–12.35
4	–37.64	–15.80	4.39	–11.00
3	–34.74	–11.81	1.82	–12.15
10	–29.16	–13.63	2.09	–6.76
5	–27.69	–14.65	1.25	–4.42
2	–25.97	–13.45	2.31	–4.92
7	–24.46	–12.79	1.96	–4.41

### 3.3 DNA Cleavage study

**Figure 7:**
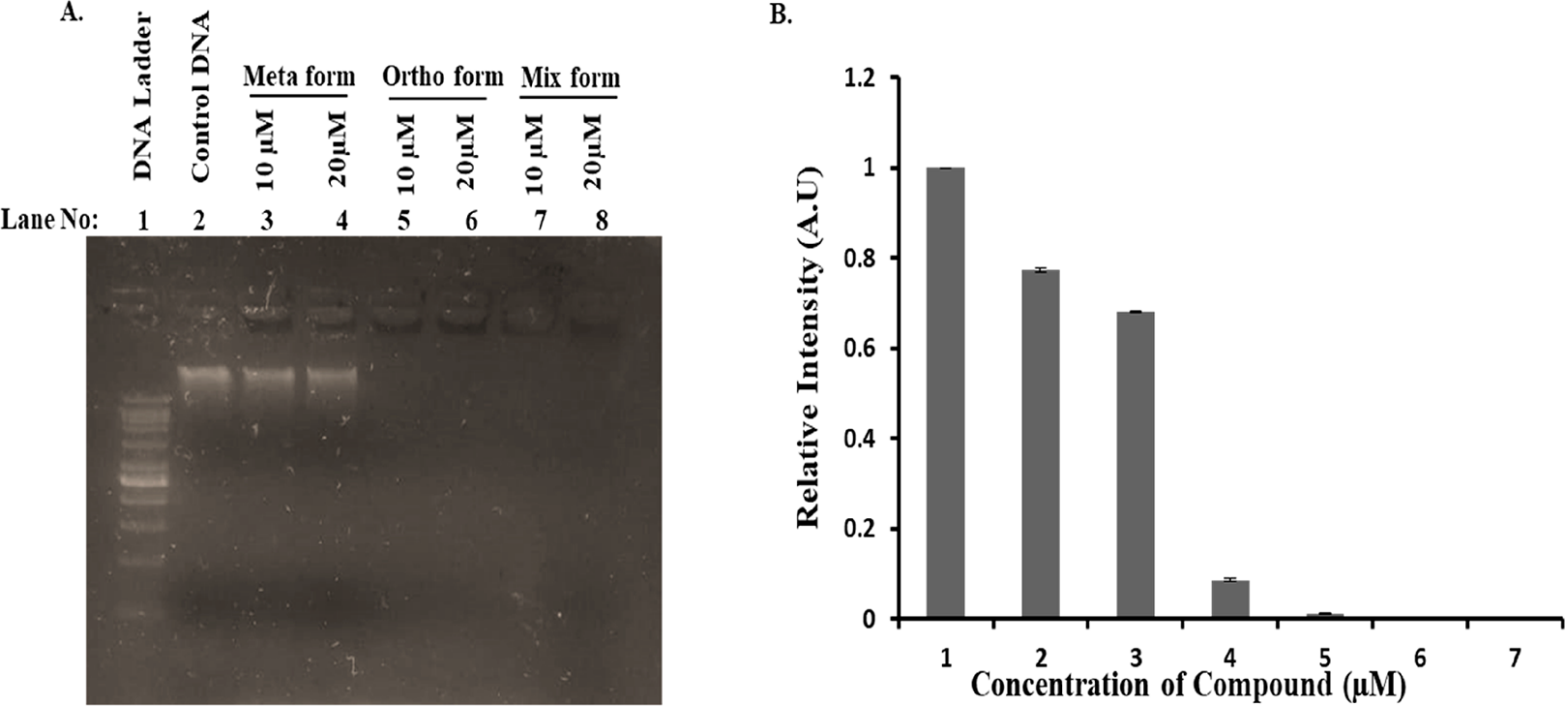
Nuclease activity of meta, ortho, and mixed forms of compounds. The DNA cleavage study of meta, ortho, and mixed forms of compounds was performed on 1% agarose gel and visualized under UV light (A). The extent of DNA cleavage was measured by ImageJ software and represented in graphical form (B).

DNA cleavage studies of meta, ortho, and mixed forms of compounds were performed against *E. coli* genomic DNA in a dose-dependent manner. The DNA cleavage study showed that ortho and mixed forms (lanes 5–6 and 7–8 respectively, Figure 7) of compounds at concentrations 10 and 20 μM exhibit nuclease activity compared to untreated DNA and the meta form (lanes 2 and 3–4, respectively, Figure 7). The DNA ladder was applied as a reference in agarose gel (lane 1) and to estimate the size of cleaved DNA. Thus, from these findings, we can conclude that the compounds inhibit the growth and replication of *E. coli* by cleaving the genome.

## 4 Conclusion

Polymers of chloroaniline have been synthesized successfully, and the ordered structure of polymer has been successfully examined by powder X-ray diffraction pattern. UV–visible spectrum suggested the π–π* transition in the phenyl ring and n-π* excitation of benzenoid to quinoid moieties. SEM images showed the branching of the polymer chain. The ortho and mixed form inhibits the nuclease activity and retards the growth and replication of *E. coli*. The compounds were found to be effective in preventing *E. coli* from proliferating and replicating. The molecular docking studies followed by biophysical interaction study showed the strong binding of poly-*m*-chloroaniline with both the targets HTIIIbTB and SAGS in comparison to the ortho and mixed forms.

## Data Availability

Not Applicable.
